# Myxoid stroma is associated with postoperative relapse in patients with stage II colon cancer

**DOI:** 10.1186/s12885-020-07335-w

**Published:** 2020-09-03

**Authors:** Takashi Okuyama, Shinichi Sameshima, Emiko Takeshita, Takashi Mitsui, Takuji Noro, Yuko Ono, Tamaki Noie, Shinichi Ban, Masatoshi Oya

**Affiliations:** 1 Department of Surgery, Saitama Medical Center, Dokkyo Medical University, 〒 343-8555 2-1-50 Minami-Koshigaya, Koshigaya, Saitama Japan; 2 Department of Pathology, Saitama Medical Center, Dokkyo Medical University, Saitama, Japan

**Keywords:** Myxoid stroma, Desmoplastic reaction, Stage II colon cancer

## Abstract

**Background:**

Fibrosis surrounding cancer cells has been shown to affect cancer cell metastatic behavior. The present study aimed to explore the utility of myxoid stroma as a predictive factor for postoperative relapse in patients with stage II colon cancer.

**Methods:**

The present study retrospectively investigated 169 patients who underwent curative surgical resection of stage II colon cancer. The fibrotic stroma was classified according to Ueno’s criteria, and the patients were divided into the myxoid (MY) group and the non-MY (NMY) group. We also recorded tumor budding (TB) and investigated the combination of MY and TB for postoperative relapse. Postoperative survival was also explored.

**Results:**

Thirty-two (18.9%) patients had MY. MY was significantly associated with tumor budding (TB) and postoperative relapse (*p* <  0.001 and *p* <  0.001, respectively). The 5-year RFS rates in MY group and NMY group were 52.1 and 94.6% (*p* < 0.0001), and the 5-year OS rates in MY group and NMY group were 74.6 and 93.3% (*p* = 0.001). Multivariate analysis showed that both MY and TB were significant risk factors for postoperative relapse (*p* < 0.001 and *p* = 0.02, respectively), and that only TB was a significant risk factor for OS (*p* = 0.043). Furthermore, compared with patients with either one of MY or TB, patients with both MY and TB had postoperative relapse more frequently (11.4% vs. 53.8%).

**Conclusions:**

The present study suggests that MY is a predictive marker for postoperative relapse in patients with stage II colon cancer.

## Background

The actual role of adjuvant chemotherapy (AC) in patients with stage II colon cancer remains unclear despite several clinical trials and meta-analyses [1–4]. However, surveillance, epidemiology, and end results analyses have shown that there are groups of patients having a higher risk of relapse than others [5]. International guidelines therefore recommend that patients with stage II colon cancer should be divided into a high-risk group and a low-risk group for postoperative relapse, and that AC should be considered in patients with high-risk features, but high-level evidence including molecular and genetic factors is still lacking [6–12].

Current molecular and genetic studies have indicated that tumor progression, growth, and spread are determined by the interaction between a cancer and its surrounding stromal cells [13, 14]. The tumor stroma contains many different cells, including lymphocytes, macrophages, leukocytes, Rouget cells, vascular endothelial cells, and fibroblasts. Of these stromal cells, it is increasingly clear that active fibroblasts (cancer-associated fibroblasts, CAFs) are an important regulator of cancer progression, invasion, and metastasis [15, 16].

CAFs or myofibroblasts induce peritumoral fibrosis that may promote tumoral progression by regulating the interaction between cancer and stromal cells [17, 18]. The fibrotic stroma (i.e. desmoplastic reaction, DR) refers to the state of extracellular matrix (ECM) remodeling generated by CAFs in cancer stroma [19, 20]. Recent reports have indicated a close relationship between the form of DR and the prognosis of patients with several solid cancers [21–23]. In the present study, the aim was to explore the relationship between myxoid stroma (one of the forms of DR) and postoperative relapse in patients with stage II colon cancer.

## Methods

Resected specimens from 169 consecutive patients with stage II colon cancer at Saitama Medical Center, Dokkyo Medical University between April 2010 and March 2016 were included in this study. Patients who received neoadjuvant chemotherapy and patients with synchronous or metachronous, multiple advanced cancers were excluded. Pathological findings were classified according to the TNM classification of Malignant Tumors (8th edition) [24]. The lymph node dissection was considered sufficient when 12 or more lymph nodes had been examined pathologically [24]. This study was approved by the Ethics Committee of Saitama Medical Center, Dokkyo Medical University (No. 1762).

### Desmoplastic reaction and tumor budding

**Fig. 1:**
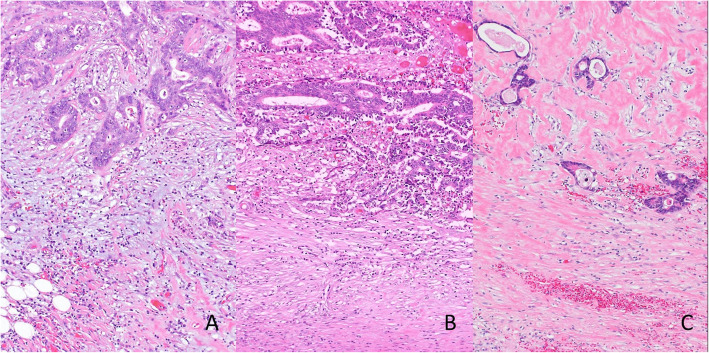
**a** Immature type DR: amorphous and myxomatous stroma with slightly basophilic matrix. **b** Intermediate type DR: keloid-like, thick bundles of hypocellular collagen with bright eosinophilic hyalinization around the tumor nests. **c** Mature type DR: fine mature collagen fibers stratified into multiple layers. Fibrotic stroma contains neither myxomatous stroma nor keloid-like collagen (**a**, **b**, **c**, hematoxylin and eosin, Magnifications × 100). DR, desmoplastic reaction

DR was assessed at the advancing edge of a primary tumor with hematoxylin and eosin (H&E) staining, and the specimens were divided according to Ueno’s criteria: the myxoid stroma (MY) group and the non-myxoid stroma (NMY) group [25] (Fig. 1). Immature stroma in Ueno’s criteria was defined as MY, and mature fibrosis and keloid-like stroma was defined as NMY in this study. When tumors involved the areas of myxoid changes (an amorphous stromal substance with basophilic extracellular material), the DR was recorded as MY regardless of the existence of mature stroma or keloid-like collagens. Small areas of myxoid fibrotic change were assessed according to whether these features were observed with a 40× objective lens in areas without microscopic abscesses.

DR was evaluated by two independent observers. One of them was an experienced pathologist (YO), with no knowledge of outcomes and other clinical information, and the other was a surgeon (TO). Their inter-observer agreement was calculated using *k*-statistics. The inter-observer agreement coefficient *k* was 0.72.

Tumor budding (TB) was evaluated with H&E staining and defined as single cells or clusters of up to four cells at the advancing edge of colorectal cancer [26]. The evaluation of TB was performed under a 20× objective lens in the areas where TB was observed most frequently (hotspot method) [27]. TB was classified as low-grade (0–4 buds), intermediate-grade (5–9 buds), and high-grade (10 or more buds) [27, 28].

### Follow-up and adjuvant chemotherapy

Postoperative surveillance including medical examinations and laboratory tests was performed every 3 months, and computed tomography (CT) of the chest, abdomen, and pelvis was performed every 6 months until 5 years after surgery. Surveillance colonoscopy was performed within 1 year after surgery and annually thereafter until no neoplastic lesions were observed. If no neoplastic lesions were detected on surveillance colonoscopy, the subsequent colonoscopy was performed 3 years later. Adjuvant chemotherapy (AC) was usually recommended to patients with high-risk features, such as T4 lesions, poor differentiation, lymphovascular or perineural invasion, < 12 lymph nodes harvested, positive margin, or bowel obstruction or perforation, for a total of 6 months using a regimen including 5-fluorouracil (5-FU)-leucovorin or capecitabine (FOLFOX or CAPOX). The use and selection of the PAC regimen depended on the primary surgeon.

### Statistical analysis

Associations between MY and clinicopathological findings were assessed using the two-tailed chi-squared test or Fisher’s exact test. Continuous variables were assessed using the Mann-Whitney U test. RFS rate was calculated from the date of surgery to the date of diagnosis of tumor relapse. OS rate was calculated from the date of surgery to the date of death by any cause or the last follow-up visit. RFS and OS rates were calculated using the Kaplan-Meier method, and differences were evaluated using the log-rank test. Multivariate analysis was performed using the Cox proportional hazard regression model with data that trended toward significance (*P* < .10) on univariate analysis. A two-sided *P* value of <.05 was considered significant. All analyses were performed using the SPSS statistical software package, version 24 (IBM Japan Ltd., Tokyo, Japan).

## Results

### Patients’ clinical and pathological characteristics

More than half of the patients were men (55.6%), and the patients’ average age at the time of surgery was 70.5 years (range 29–92 years). The median follow-up period of the entire patient group was 60 months. The baseline clinicopathological characteristics of the 169 patients are shown in Table 1. In this study, 32 patients (18.9%) had MY (MY group), and 137 (71.1%) patients did not (NMY group). The regimens of adjuvant chemotherapy in MY group and NMY group are shown in Table 2. Patients who received completely AC was 22 (13.6%) cases (MY; 6 patients, NMY; 16 patients). Three of 22 patients who were withdrawal due to have a drug allergy, were excluded from AC group. These patients received 2–4 cycles intravenous or oral AC. Twenty-three of 169 patients developed postoperative relapses, including lung metastasis (6 patients), liver metastasis (8 patients), peritoneal dissemination (5 patients), metastasis to the para-aortic lymph nodes (3 patients), and local relapse (1 patient). Seventeen patients (17/169, 10.1%) died of any causes within the follow-up period, and seven of these patients (7/17, 41.2%) died of causes other than primary colon cancer.

**Table 1:** Demographic and clinical characteristics, postoperative treatment, and outcome at baseline of participants in this study

Characteristics	Number (%)	Characteristics	Number (%)
Gender		Surgical margin	
Male	94 (55.6)	Negative	163 (96.4)
Female	75 (44.4)	Positive	6 (3.6)
Age (yr, average, range)	70.5 (29–92)	Glucose tolerance	
Preoperative serum CEA value		Normal	120 (71.0)
< 5	106 (62.7)	Abnormal	49 (29.0)
≥ 5	63 (37.3)	Obstruction / perforation	
Preoperative serum CA19–9 value		Absence	157 (92.9)
< 37	143 (84.6)	Presence	12 (7.1)
≥ 37	26 (15.4)	Surgical approach	
Tumor location		Laparotomy	101 (59.8)
Right	88 (52.1)	Laparoscopic surgery	68 (40.2)
Left	81 (47.9)	Examined lymph node	
Tumor size (cm, average, range)	5.0 (1–17)	≥ 12	115 (68.0)
Differentiation		< 12	54 (32.0)
Well, Mod, pap	150 (88.8)	Postoperative adjuvant chemotherapy	
Por, Muc, Sig	19 (11.2)	No	147 (86.1)
pT category		Yes	22 (13.9)
pT3	144 (85.2)	Postoperative relapse	*n* = 23
pT4a	13 (7.7)	Lung	6
pT4b	12 (7.1)	Liver	8
Lymphatic invasion		Peritoneum	5
Absent	104 (61.5)	Paraaortic lymph node	3
Present	65 (38.5)	Local relapse	1
Vessel invasion		Desmoplastic reaction	
Absent	71 (42.0)	Mature stroma	76 (45.0)
Present	98 (58.0)	Intermediate stroma	62 (36.1)
Tumor budding		Myxoid (i.e. immature) stroma	32 (18.9)
Grade 1	106 (62.7)		
Grade 2	43 (25.4)		
Grade 3	20 (11.9)

**Table 2:** The regimens of adjuvant chemotherapy used in patients with or without myxoid stroma

Adjuvant Chemotherapy						
	No	UFT/LV	CAPE	FOLFOX	XELOX	Withdrawal
NMY	118	6	5	3	2	3
MY	26	3	1	2	0	0

*UFT/LV* Fluorouracil plus leucovorin, *CAPE* Capecitabin, *CAPEOX* CAPE plus oxaliplatin, *FOLFOX* Fluorouracil, leucovorin plus oxaliplatin

### Relationships between myxoid stroma and clinicopathological characteristics

The relationships between MY and clinicopathological characteristics are shown in Table 3. TB and postoperative relapse were significantly more frequent in the MY group than in the NMY group (*p* < 0.001 and *p* < 0.001, respectively). None of the other clinicopathological characteristics was associated with the presence of MY.

**Table 3:** The relationships between the myxoid stroma and clinicopathological characteristics analyzed using χ^2^ and Fisher’s exact tests

	Myxoid				Myxoid		
	Absent (%)	Present (%)	***p*** value		Absent (%)	Present (%)	***p*** value
Gender)				Lymphatic invasion			
Female	62 (45)	13 (41)	0.7	Absent	87 (64)	17 (53)	0.36
Male	75 (55)	19 (59)		Present	50 (36)	15 (47)	
Age				Vessel invasion			
< 70	53 (39)	16 (50)	0.32	Absent	61 (45)	10 (31)	0.23
≥ 70	84 (61)	16 (50)		Present	76 (55)	22 (69)	
Serum CEA value				Tumor budding			
< 5	85 (62)	21 (66)	0.84	Grade 1	100 (73)	6 (19)	< 0.001
≥ 5	52 (38)	11 (34)		Grade 2, 3	37 (27)	26 (81)	
Serum CA19–9 value				Surgical margin			
< 37	116 (85)	27 (84)	0.26	Negative	133 (97)	31 (97)	1*
≥ 37	21 (15)	5 (16)		Positive	4 (3)	1 (3)	
Tumor location				Examined lymph node			
Right	72 (53)	16 (50)	0.85	< 12	42 (31)	12 (38)	0.53
Left	65 (47)	16 (50)		≥ 12	95 (69)	20 (62)	
Tumor size (cm)				Obstruction/perforation			
< 5	63 (46)	18 (56)	0.33	Absent	128 (93)	29 (91)	0.7*
≥ 5	74 (54)	14 (44)		Present	9 (7)	3 (9)	
Differentiation				Glucose tolerance			
Well, moderately, pap	121 (88)	29 (91)	1*	Normal	95 (69)	25 (78)	0.39
Por, Muc, Sig	16 (12)	3 (9)		Abnormality	42 (31)	7 (22)	
Tumor depth				Tumor relapse			
pT3	118 (86)	26 (81)	0.58	Absent	129 (94)	17 (53)	< 0.001
pT4	19 (14)	6 (19)		Present	8 (6)	15 (47)	

*Fisher’s exact tests

### Survival analyses of relapse-free survival and overall survival

**Fig. 2:**
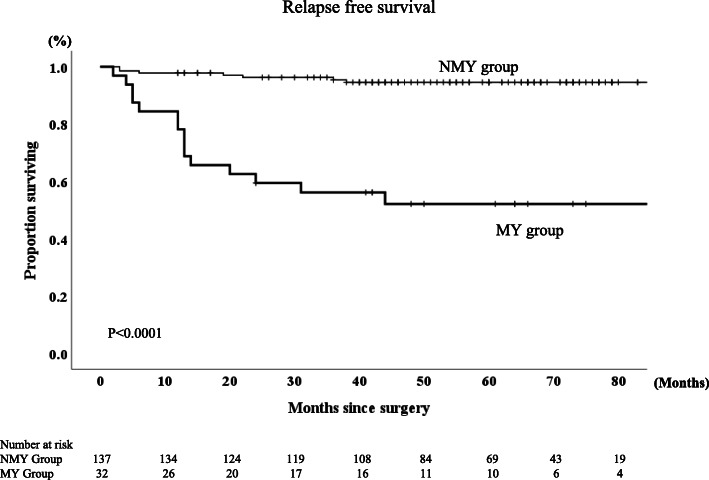
Relapse-free survival rates in NMY group and MY group

**Fig. 3:**
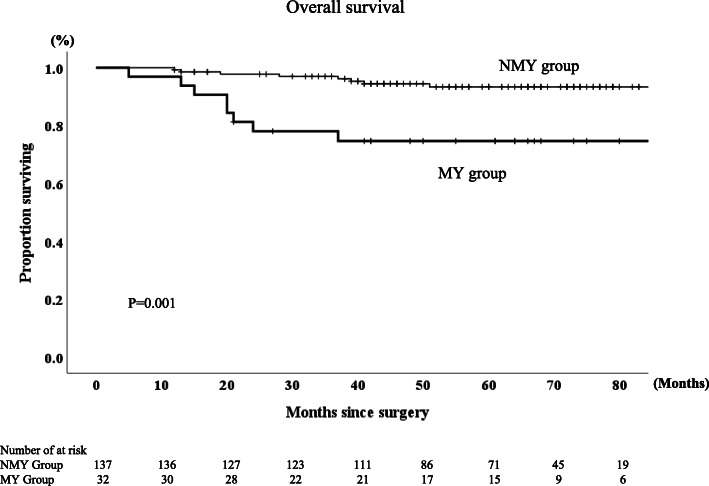
Overall survival rates in NMY group and MY group

**Fig. 4:**
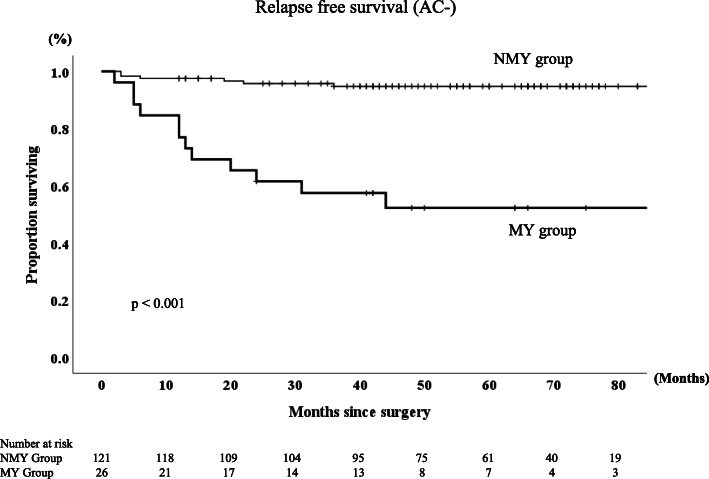
Relapse-free survival rates in NMY group and MY group without adjuvant chemotherapy

**Fig. 5:**
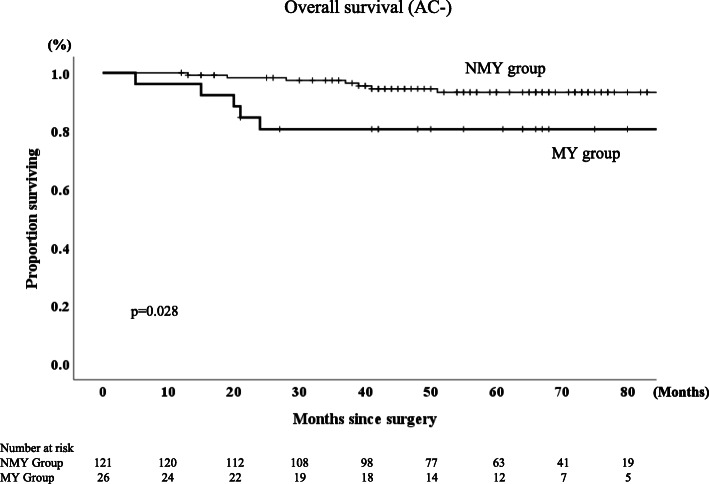
Overall survival rates in NMY group and MY group without adjuvant chemotherapy

The 5-year RFS and OS rates were 87 and 89.6%, respectively, for the entire group of patients in this study. The MY group had significantly lower RFS and OS rate than the NMY group (*p* < 0.0001 and *p* = 0.001, respectively, Figs. 2, 3). The 5-year RFS rates in MY group and NMY group were 52.1 and 94.6% (*p* < 0.0001), and the 5-year OS rates in MY group and NMY group were 74.6 and 93.3% (*p* = 0.001). Regarding patients who did not receive AC, the MY group had significantly lower RFS and OS rates than the NMY group (*p* < 0.001 and *p* = 0.028, respectively, Figs. 4 and 5).

### Univariate- and multivariate analyses to identify characteristics related to relapse-free survival and overall survival

Univariate and multivariate analyses to identify characteristics significantly related to RFS and OS rates were performed using Cox proportional hazard models (Table 4). On univariate analyses for RFS rate, TB, surgical margin, and the presence of MY were significantly associated with lower RFS rate (*p* < 0.001, *p* = 0.038 and *p* = < 0.001, respectively). Regarding the OS rate, the serum CA19–9 level, deep tumor penetration, TB, positive surgical margin, and the presence of MY were significantly associated with lower OS rate in univariate analysis (*p* = 0.026, *p* = 0.011, *p* = 0.001, *p* = 0.014 and *p* = 0.003).

**Table 4:** Uni- and multi-variate analyses using Cox proportional hazard regression models to identify to Clinicopathological characteristics related to relapse free survival and overall survival

	RFS						OS					
Predictor	Univariate analysis			Multivariate analysis			Univariate analysis			Multivariate analysis		
	HR	95%CI	***p*** value	HR	95%CI	***p*** value	HR	95%CI	***p*** value	HR	95%CI	***p*** value
Gender												
Male	1						1.00					
Female	0.71	0.298–1.697	0.44				0.67	0.247–1.808	0.43			
Age												
< 70 yr	1						1.00					
≥ 70 yr	1.05	0.446–2.445	0.92				2.43	0.792–7.465	0.12			
Serum CEA level												
< 5	1						1.00					
≥ 5	1.41	0.609–3.264	0.42				0.91	0.337–2.465	0.86			
Serum CA19–9 level												
< 37.5	1						1.00			1		
≥ 37.5	1.73	0.638–4.693	0.28				3.11	1.149–8.428	0.026	0.62	0.355–1.096	0.10
Tumor location												
Right	1						1.00					
Left	1.10	0.478–2.541	0.82				0.58	0.214–1.565	0.28			
Differentiation												
Well, moderately, pap	1						1.00					
por, muc	0.36	0.049–2.698	0.32				1.18	0.268–5.198	0.83			
Tumor size												
≤ 5	1						1.00					
> 5	1.22	0.536–2.789	0.63				0.80	0.310–2.080	0.65			
Tumor depth												
pT3	1			1			1.00			1		
pT4	2.52	0.986–6.452	0.054	1.75	0.594–5.153	0.31	3.66	1.352–9.922	0.011	0.84	0.429–1.651	0.62
Lymphatic invasion												
Absent	1						1.00			1		
Present	1.03	0.398–2.264	0.91				2.48	0.941–6.515	0.07	0.91	0.519–1.596	0.74
Vessel invasion												
Absent	1						1.00					
Present	1.65	0.674–4.056	0.27				1.09	0.416–2.875	0.86			
Tumor budding												
Grade 1	1			1			1.00			1		
Grade 2,3	9.00	3.042–26.624	< 0.001	4.11	1.301–13.002	0.02	6.18	2.015–18.977	0.001	1.94	1.020–3.668	0.043
Surgical margin												
Absent	1			1			1.00			1		
Present	4.69	1.088–20.207	0.038	4.33	0.769–24.343	0.10	6.52	1.465–28.999	0.014	0.56	0.219–1.411	0.22
Lymph node yield												
12 or more	1						1.00					
Less than 12	1.03	0.420–2.524	0.95				1.10	3.87–3.131	0.86			
Obstruction/perforation												
Absent	1						1.00					
Present	1.39	0.325–5.945	0.66				1.85	0.423–8.110	0.41			
Operation												
Laparotomy	1						1.00					
Laparoscopic surgery	1.26	0.542–2.906	0.60				1.13	0.427–2.972	0.81			
Glucose tolerance												
Normal	1						1					
Abnormality	1.88	0.636–5.556	0.25				1.92	0.550–6.677	0.31			
Adjuvant chemotherapy												
No	1						1					
Yes	1.07	0.317–3.622	0.91				2.18	0.710–6.683	0.17			
Myxoid stroma												
Absent	1			1			1			1		
Present	11.24	4.572–27.613	< 0.001	6.80	2.579–17.911	< 0.001	4.14	1.596–10.742	0.003	0.66	0.371–1.157	0.15

On multivariate analysis for RFS and OS rates, the presence of MY and TB were significant risk factors for RFS rate (MY: HR, 6.80; 95% CI, 2.579–17.911; *p* < 0.001, TB: HR, 4.11; 95% CI, 1.301–13.002; *p* = 0.02), and only TB was a significant risk factor for OS rate (HR, 1.94; 95% CI, 1.020–3.668; *p* = 0.043).

### Combination of myxoid stroma and tumor budding for postoperative relapse and survival

The relationships of the combination of MY and TB with 5-year RFS and OS rates are shown in Table 5. The relapse rate in patients with both MY and TB was nearly five-fold higher than with either one of MY or TB (53.8% vs. 11.4%). Patients with both MY and TB had a much worse 5-year RFS rate than those with either one of MY or TB (44.2% vs. 87.7%, *p* < 0.001). Furthermore, Cox regression analysis showed that there was a significant difference in RFS rate between patients with both MY and TB and those with either one of MY or TB (HR, 5.801; 95% CI, 2.0884–16.146; *p* = 0.001). Regarding the OS rate, there was no significant difference between patients with both MY and TB and those with either one of MY or TB on Cox regression analysis.

**Table 5:** Analyses using Cox proportional hazard model for the combination myxoid stroma and tumor budding on relapse free survival and overall survival rates

Combinations	Number of relapse (%)	5-year RFS rate (%)	HR	95% CI	*p* value	5-year OS rate (%)	HR	95% CI	*p* value
MY- / TB -	4/99 (4)	96.7	1			97.9	1		
MY - / TB +	4/37 (10.8)	88.1	4.003	0.896–17.896	0.069	85.2	8.75	1.766–43.360	0.008
MY+ / TB -	1/7 (14.3)	85.7	4.798	0.499–46.134	0.174	71.4	12.95	1.815–92.432	0.011
MY+ / TB +	14/26 (53.8)	44.2	24.42	6.992–85.255	< 0.001	72.5	15.64	3.245–75.337	0.001
MY+ / TB- or MY- / TB+	5/44 (11.4)	87.7	1			82.1	1		
MY+ / TB +	14/26 (53.8)	44.2	5.801	2.084–16.146	0.001	72.5	1.619	0.596–4.562	0.335

*MY* Myxoid stroma, *TB* Tumor budding

## Discussion

The aim of the present study was to explore the utility of MY as a predictor of postoperative relapse and survival in patients with stage II colon cancer, respectively. In addition, the utility of the combination of MY and TB was similarly explored. In this study, patients with MY had more frequent postoperative relapse than those without MY. Furthermore, Kaplan-Meier analyses showed that patients with MY had significantly worse RFS and OS rates than those without MY, and multivariate analysis demonstrated that MY was an independent predictor of postoperative relapse in patients with stage II colon cancer. On the analysis for the combination of MY and TB, patients with both MY and TB had a significant worse 5-year RFS rate than those with either one of MY or TB. These results suggest a close relationship between MY and cancer cell metastatic behavior.

DR classified by the type of fibrotic stroma is simply detected by routine H&E staining. This inexpensive pathological feature has been reported as a useful predictor for metastasis and survival in several solid cancers, including cutaneous cancer, breast cancer, lung cancer, pancreatic cancer, and colorectal cancer [21, 29–32]. In colorectal cancer, although the amount of fibrosis was evaluated as an index of the stromal reaction first, Ueno et al. recently have proposed categorizing it into three types by the fibrotic responses of cancer stroma, and they reported that their classification was useful for predicting the prognosis of patients with colorectal cancer [32–34].

Recent approaches using molecular and genetic methods may offer predictive information for relapse and survival in patients with stage II colon cancer in addition to the routine pathological examination [9, 10]. Microsatellite instability (MSI) due to mutation or modification of mismatch repair (MMR) is mainly detected in Lynch syndrome, which is an autosomal dominant cancer syndrome associated with an increased risk of colorectal cancer and other extracolonic malignancies, including endometrial cancer, stomach, small bowel, and ovarian cancers [35, 36]. In stages II/III colorectal cancer, it is generally recognized the biological difference in tumors with MMR states [9, 10, 37]. Hutchins et al. demonstrated that patients with dMMR had significantly lower relapse than that with pMMR [38]. On the other hand, Ribic et al. and Sargent et al. reported that there was no benefit from FU based AC in stage II/III patients with dMMR [37, 39]. In addition, Gavin et al. showed MMR states were not predictive for oxaliplatin benefit [10]. Although MSI or the MMR test need immunohistochemistry or expensive gene analyses, clinical evidence supporting their use is still insufficient.

Among cells of the stromal component, fibroblasts have been termed cancer-associated fibroblasts (CAFs) and are important promotors of tumor progression and metastasis [40]. CAFs can induce dedifferentiation by extracellular matrix (ECM) remodeling and desmoplasia by increasing the deposition of ECM [20, 41]. In several reports, single or small clusters of cancer cells (TB) have been frequently observed at MY areas [25, 29], and TB was associated with DR in the present study. These results suggest that CAFs may have a critical role in regulating DR and TB. Recently, Ueno et al. reported that a tumor grading system based on DR and TB provides more precise prediction of individual patients than the conventional staging [42]. In the present study, compared with patients with either one of MY or TB, those with both MY and TB developed postoperative relapse much more frequently. The presence of both MY and TB might have a synergistic effect rather than an additive effect for postoperative relapse.

Circulating CAFs (cCAFs) have recently been identified in the circulation, and metastasis-associated fibroblasts at the metastatic site promote the proliferation of cancer cells [43, 44]. Cornil et al. reported that fibroblasts affected the proliferation of melanoma cells, and that only metastasized melanoma cells were influenced by fibroblasts [45]. Since MY has been closely associated with metastasis in previous reports [21, 29–32], specific stimuli released by MY may move cCAFs to metastatic sites. In fact, 65% of patients with MY developed tumor relapse, whereas only 12% of patients without MY did.

The present study has several limitations, such as its retrospective design and small sample size in a single institution. A difference in the relapse rate between patients with mature stroma and those with intermediate stroma might have been found if the sample size had been larger. In the present study, multivariate analysis did not show that MY was an independent risk factor for OS in patients with stage II colon cancer. This might have been due to the short follow-up and the effects of AC and treatment for relapse, such as surgical intervention and intensive chemotherapy. In addition, neither MSI nor MMR, which are considered to be predictive in the NCCN guideline, was examined, and the effect of AC according to the NCCN guideline was not examined.

In conclusion, the present results suggest that MY is a useful predictive feature for postoperative relapse in patients with stage II colon cancer. Although the additional larger trials should be added, if both MY and TB are identified in the lesion, the risk of tumor relapse may be a high. Since MY may become a useful information for deciding whether to add AC in patients with stage II colon cancer, it should be confirmed in multi-institutional trials for a large number of patients.

## Data Availability

The datasets used and/or analyzed during the current study are available from the corresponding author on reasonable request.

## Abbreviations

AC: Adjuvant chemotherapy
CAF: Cancer-associated fibroblast
CAPE: Capecitabin
CAPEOX: CAPE plus oxaliplatin
DR: Desmoplastic reaction
FOLFOX: Fluorouracil, leucovorin plus oxaliplatin
MA: Mature stroma
ECM: Extracellular matrix
IN: Intermediate stroma
MSI: Microsatellite instability
MMR: Mismatch repair
MY: Myxoid stroma
NCCN: National Comprehensive Cancer Network
NMY: Non-myxoid stroma
UFT/LV: Fluorouracil plus leucovorin
OS: Overall survival
RFS: Relapse-free survival
TB: Tumor budding
